# Impact of Lateral Transfers on the Genomes of Lepidoptera

**DOI:** 10.3390/genes8110315

**Published:** 2017-11-09

**Authors:** Jean-Michel Drezen, Thibaut Josse, Annie Bézier, Jérémy Gauthier, Elisabeth Huguet, Elisabeth Anne Herniou

**Affiliations:** Institut de Recherche sur la Biologie de l’Insecte, UMR CNRS 7261, UFR des Sciences et Techniques, Université de Tours-François Rabelais, 37200 Tours, France; thibaut.josse@univ-tours.fr (T.J.); annie.bezier@univ-tours.fr (A.B.); mr.gauthier.jeremy@gmail.com (J.G.); elisabeth.huguet@univ-tours.fr (E.H.); elisabeth.herniou@univ-tours.fr (E.A.H.)

**Keywords:** horizontal gene transfer, endogenous viruses, transposable elements, gene domestication, baculovirus, polydnavirus, lepidoptera, hymenoptera

## Abstract

Transfer of DNA sequences between species regardless of their evolutionary distance is very common in bacteria, but evidence that horizontal gene transfer (HGT) also occurs in multicellular organisms has been accumulating in the past few years. The actual extent of this phenomenon is underestimated due to frequent sequence filtering of “alien” DNA before genome assembly. However, recent studies based on genome sequencing have revealed, and experimentally verified, the presence of foreign DNA sequences in the genetic material of several species of Lepidoptera. Large DNA viruses, such as baculoviruses and the symbiotic viruses of parasitic wasps (bracoviruses), have the potential to mediate these transfers in Lepidoptera. In particular, using ultra-deep sequencing, newly integrated transposons have been identified within baculovirus genomes. Bacterial genes have also been acquired by genomes of Lepidoptera, as in other insects and nematodes. In addition, insertions of bracovirus sequences were present in the genomes of certain moth and butterfly lineages, that were likely corresponding to rearrangements of ancient integrations. The viral genes present in these sequences, sometimes of hymenopteran origin, have been co-opted by lepidopteran species to confer some protection against pathogens.

## 1. Introduction

Although they attract most investigations from biologists, functional genes only constitute a minor part of genome contents, which contain a high proportion of viruses or remnants of genetic parasites. Acquisition of this DNA have most often involved at some point Horizontal Gene Transfer (HGT), which is defined as the accidental acquisition of DNA from another species, independently of reproduction, and regardless of the evolutionary distance between the two species. Evidence that HGT occurs not only in bacteria or unicellular eukaryotes, but also in multicellular organisms has been accumulating in the past few years [1,2,3,4]. However, the extent of this phenomenon remains controversial: on the one hand, the false identification of HGT resulting from the contamination of DNA sequenced leads to overestimation of HGT frequency, on the other hand, filters excluding, *a priori*, foreign sequences from genome assembly leads to their underestimation [5]. The majority of reported HGT events between metazoans concern transposable elements (TEs), which are selfish DNA sequences that are capable of excising or copying themselves from one genomic locus to integrate into another locus, generally within the same genome. From time to time, these elements enter a new genome, where they can spread until their expansion becomes regulated by the new host [6].

In addition to mobile elements, remnants of virtually all the virus families, the so-called endogenous viral elements (EVEs), can also be found in invertebrate genomes [7,8,9]. This could be expected from viruses that integrate their genome into that of their host as an essential step of their life cycle, like retroviruses. However, many EVEs derive from the viruses displaying no DNA stage, and it appears that all kinds of viruses end up in genomes, although the mechanisms involved are generally unknown [7,9,10]. Most of the time, after their integration, EVEs slowly decay under neutral molecular evolution, because they are no longer active. In some cases, EVEs may however provide a new function to the host [11]. DNA sequences from the endosymbiont, *Wolbachia*, which is widespread in arthropods, and particularly present within germinal cells, have also been found in the genomes of many invertebrate species [12]. Again, most transferred *Wolbachia* genes are fossils embedded in host genomes. However a recent study has reported a case showing they might interfere with sex determination [13].

Besides selfish and pathogenic DNA, HGTs have mediated the acquisition of several genes conferring new functions to vertebrates and invertebrates [2]. Viruses may act as vehicles for such transfers, as they can pick up genes or TEs from the cells they infect, and transfer them to their next hosts [14]. However, viruses generally transfer genes between the related eukaryote species that they infect. There are many reported examples of ancient acquisitions of genes of prokaryotic origin, in nematodes and insects, thus allowing these organisms to feed on plants using cell wall degrading enzymes [15,16]. In these cases, the mechanisms of transfer that are involved are unclear. Moreover, in order to provide a new physiological function to their recipient species, the genes have to adapt to the eukaryotic expression machinery, which is different from that of prokaryotes.

**Figure 1:**
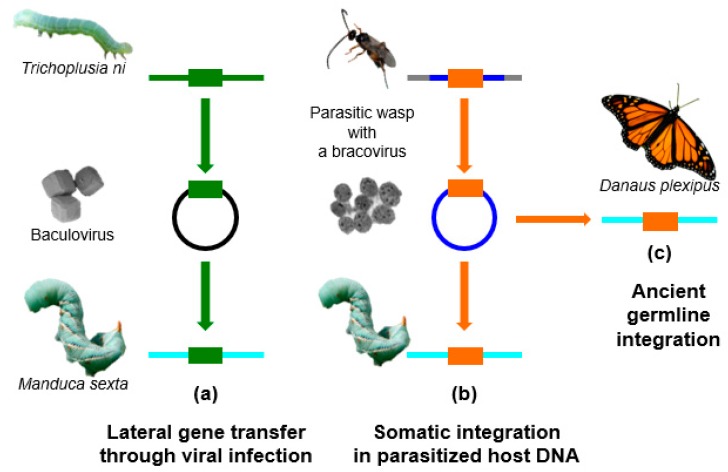
Virus mediated-transfers in Lepidoptera. (**a**) Baculoviruses pick up genes from the lepidopteran species they infect, and can probably transfer genes or TEs to other species belonging to their host range. (**b**) The endogenous bracovirus associated with parasitoid wasps produces particles, and the DNA circles enclosed in the particles integrate into parasitized host cells as part of the bracovirus life cycle. (**c**) The presence of bracoviral sequences fixed in the genomes of several Lepidoptera lineages suggests that, from time to time, bracovirus circles may also integrate into the DNA of germline cells. Bracoviral DNA insertions might confer a selective advantage to the individuals producing certain bracovirus gene products, such as a protection against other viruses. Colored rectangles represent transferred genes, colored lines represent genomic DNA of the species represented, the circles correspond respectively to a baculovirus genome (**a**) and to a bracovirus circle packaged in the particles (**b**). Picture credits A. Bézier, J. Gaillard & J. Herbinière.

Here, we will review the current advances on HGTs, with a more specific focus on Lepidoptera. In the past few years, many genomes of Lepidoptera have been sequenced, allowing the identification of foreign DNA within these sequences. It appears that this insect order has been particularly impacted by HGTs, possibly because of associated viruses and parasites. In particular, a deep sequencing approach on the DNA contained in infectious virus particles showed that lepidopteran genomes are regularly exposed to TEs or other host sequences during infections by large DNA viruses from the *Baculoviridae* family. For example, baculoviruses pick up genes from the lepidopteran species that they infect, and can probably transfer genes or TEs to other species belonging to their host range (Figure 1a). Recent studies have also shown that some EVEs derived from nudiviruses [17] (a family of large DNA viruses infecting insects and crustaceans) have also been involved in HGT events targeting Lepidoptera. These EVEs named bracoviruses [18,19,20,21] are harboured by tens of thousands of wasp species that parasitize lepidopteran larvae, and represent probably the most remarkable examples of endogenous viruses providing a biological function to their host. The endogenous bracovirus associated with parasitoid wasps has acquired genes from the wasp genome, which are used as virulence factors to manipulate lepidopteran larvae physiology, in order to allow wasp larvae development within the host. The DNA circles, packaged into the particles that are introduced into the host during wasp oviposition, encode these virulence factors. The circles integrate into parasitized host cells as a part of bracovirus life cycle [22] (Figure 1b). The presence of bracoviral sequences fixed in the genomes of several Lepidoptera lineages suggests that, from time to time, bracovirus circles may also integrate into the DNA of germline cells, and could be transmitted to the next generation (Figure 1c). Bracoviral DNA insertions might confer a selective advantage to the individuals producing certain bracovirus virulence factors, such as a protection against other viruses [23].

## 2. Bacterial Sequences Acquired in Lepidopteran Genomes

As described in other insect orders, numerous lepidopteran genomes have been impacted by horizontal transfers (HTs) of bacterial sequences. This phenomenon, first described in *Bombyx mori*, becomes more apparent with the increasing number of available lepidopteran genomes [24,25,26,27,28].

As in the case of other insects and arthropods, Lepidoptera can be associated with *Wolbachia*, a genus of usually facultative endosymbiontic bacteria, which infect two-thirds of insect species [29]. It was recently estimated that approximately 80% of lepidopteran species are infected with *Wolbachia,* with a reported mean prevalence of 27% [30]. *Wolbachia* most commonly causes reproductive manipulation inducing phenotypes such as feminization, male killing, and cytoplasmic incompatibility [31,32,33,34]. The fact that this bacteria can infect host germ cells probably explains why *Wolbachia* DNA integration events were detected in approximately 20% of arthropod and nematode genome sequences [12]. *Wolbachia* insertions of different sizes have been described in many insect species [35,36,37,38], with the largest insertion corresponding to nearly the entire *Wolbachia* genome in the genome of *Drosophila anassasae* [39]. HGT of *Wolbachia* sequences into lepidopteran genomes has only been investigated recently. Analysis of nine lepidopteran genomes (*Bombyx mori*, *Danaus plexippus*, *Heliconius melpomene*, *Manduca sexta*, *Melitaea cinxia*, *Papilio glaucus*, *Papilio polytes*, *Papilio xuthus,* and *Plutella xylostella*) revealed only one possible *Wolbachia* sequence transfer into the genome of the butterfly *M. cinxia* [30]. Confirmation that this sequence derives from a genuine HGT event awaits experimental validation by PCR amplification of chimeric sequences, to rule out genome missassembly and/or contamination. The *Wolbachia* sequence found in the genome of *M. cinxia* corresponds to a 350 bp portion of a gene (with over 96% identity), suggesting that the sequence is not functional. Indeed in many examples, the adaptive significance of *Wolbachia* HGTs is not evident, with many transferred genes containing mutations or displaying very low expression levels [12,35,39,40,41]. This apparent lack of evidence of frequent HGTs from *Wolbachia* to Lepidoptera could be explained by the fact that, based on divergence time estimations, *Wolbachia* may have been recently introduced in Lepidoptera [30]. Another explanation could be that *Wolbachia* sequences may have been filtered out as contaminants during genome assemblies, leaving the possibility that *Wolbachia* HGT sequences may be present in the raw sequencing reads [30].

In contrast to practical lack of evidence of *Wolbachia* HGT in lepidopteran genomes, multiple ancient HGTs involving other bacteria, and even fungi have been detected in Lepidoptera [24,25,26,27,28,42] (Table 1). In these scenarii, the horizontally transferred bacterial genes could potentially have conferred novel traits to the recipient Lepidoptera. In many instances, the microbial genes are proposed to be involved in adaptation to host plants, because they encode enzymes involved either in the assimilation of intracellular plant metabolites (carbohydrates and amino acids), or in overcoming plant defences [43]. Some of these genes are also postulated to be involved in pathogen resistance [25].

Systematic investigation of HGT events in the first sequenced lepidopteran genomes *B. mori*, *D. plexippus*, and *H. melpomene* led to the identification of 13, 12, and 12 HGTs, respectively, from bacteria and fungi [24,25,26]. All of these HGTs had homologues that are expressed in some other lepidopterans, indicating ancient transfer events [26]. Several of these transferred genes encode for β-fructofuranosidases (also called β-glucosidases), which are potentially involved in the metabolism of plant carbohydrates [44]. Comparative genomic studies revealed widespread presence of β-fructofuranosidases in Arthropods including beetles and spider mite [45,46], suggesting that multiple independant HGT events have occurred, or that an ancient HGT event occurred in an arthropod ancestor and was subsequently lost in lineages in which these genes are not present. In all of the sequenced lepidopteran genomes, horizontally transferred genes of bacterial origin coding for glycosyl hydrolase family 31 (GH31) enzymes have also been detected [26,27]. Although the function of these enzymes in Lepidoptera is not yet characterized, they are generally involved in oligosaccharide cleavage [27]. Taken together, many horizontally transferred genes of bacterial origin could represent an adaptive advantage for phytophagous lepidopterans by enabling them to metabolize plant carbohydrates.

Acquisition of horizontally transferred genes could also allow for lepidopterans to overcome plant defences. The β-fructofuranosidase gene acquired by HGT in *B. mori*, has been shown to be an active enzyme that is insensitive to inhibitory alkaloids that accumulate in mulberry plants. This suggests that the silkworm was able to adapt to mulberry plants thanks to this acquired enzymatic activity [44]. Phylogenetic analysis also indicated that Lepidoptera and mites independently acquired bacterial genes related to β-cyanoalanine synthase genes, which can detoxify cyanide potentially produced by cyanogenic plants upon herbivore attack [28,47]. These genes may have alternative functions involved in cysteine biosynthesis, which may explain why the horizontally transferred genes have been maintained in Lepidoptera that do not feed on cyanogenic plants [28]. Further comparative genomic approaches gave insight into the evolution of horizontally transferred genes [26,27]. In *B. mori*, *D. plexippus* and *H. melpomene*, post-transfer duplication of HGTs contributed to the generation of twenty or more genes in each genome [26]. Among the 14 types of HGTs that were observed in these three species, more than half had undergone several duplication events after their acquisition. The patterns of paralogues differed in the three species, suggestive of independent duplication or loss events. When combined with functional analyses, such as measuring the activity of recombinant proteins [28,47], molecular evolution data can also prove to be invaluable to make gene function predictions [26,27]. Tissue-specific expression patterns of horizontally transferred genes suggest that gene duplications lead to subfunctionalization or neofunctionalization [26]. Data revealing that three *Pieris rapae* β-cyanoalanine synthases have different activities suggest that these horizontally transferred genes may also be undergoing subfunctionalization [47]. Furthermore, change in codon bias, more conform to the DNA composition of the new genome, may allow improved expression, and therefore increase the potential of the transferred sequence to be functional. Finally, certain HGTs were shown to have evolved under purifying selection, indicating that selective forces are acting to maintain the function of these sequences [26,27].

It is predictable that growing genomic data on lepidopteran genomes, acquired via high throughput sequencing, will unveil new evidence of horizontally transferred bacterial genes, which will allow for more precise dating of HGT events, and finer studies on the evolution of the acquired genes. Furthermore, new functional approaches, allowing for targeted inactivation of acquired genes, may help to formally link acquisition of these genes with adaptive traits of lepidopteran hosts.

## 3. Transfer of Mobile Elements in Lepidoptera

Transposable elements (TEs) are a part of the genome that can transpose (i.e., move) to another location in the genome [49]. TEs are classified in two classes, depending on their transposition mechanism [50]. Class I generate RNA intermediates and move with a “copy and paste” mechanism. They are subdivided in Long Terminal Repeat (LTR) retroelements and in non-LTR retroelements, themselves constituted by Long Interspersed Nuclear Elements (LINEs) and Short Interspersed Nuclear Elements (SINEs) (SINEs are non-autonomous and rely on the transposase of another element to move). Class II generate DNA intermediates and move with a “cut and paste” mechanism [51]. TEs are important parts of genomes and contribute to their evolution [51,52]. Since a TE in a new genome first multiplies itself, it then undergoes inactivation and can finally be lost [53,54], it is now generally admitted that HTs is a part of TE life cycle [55].

The detection of horizontal transfers of TEs (termed HTTs) mostly relies on finding TE copies with high nucleotide identity in phylogenetically distant species. The first HTT cases were demonstrated between two *Drosophila* species by Daniels and collaborators [56]. To date, more than 200 HTTs are described in insects and around 5% concern HTTs in Lepidoptera [57]. Although all of the TEs are able to transfer horizontally, class II TEs constitute the majority of detected HTT events. This could be explained by the fact that the DNA intermediate of the class II TEs is more stable than the RNA intermediate of class I TEs [55,58]. Another important parameter is the requirement of host factors that are implicated in transposition. Indeed, two superfamilies of class II TEs (*Tc1/mariner* and *hAT*) are able to transpose autonomously [59], and accordingly, HTTs of these elements have been described in phylogenetically distant species [60]. In contrast, class I TEs are thought to require host factors to transpose. Thus, less class I HTTs are expected to occur, and they are more likely to affect closely related species in which the required factors should be more similar [61]. In *Drosophila*, only 4% of HTTs concern non-LTR retroelements (LINE and SINE), while 42.6% correspond to LTR retroelements HTTs and 52.4% to class II HTTs [62].

Concerning Lepidoptera, class II TEs are involved in the majority of described HTTs. Only a few HTT cases of class I TEs have been described. *CR1B*, a LINE non-LTR retroelement, has been transferred between *Bombyx* and *Maculinae* genera [63,64]. HTTs of class I SINE non-LTR retroelements also occur since the SINE *HaSE2* is found both in the Lepidoptera *Helicoverpa armigera* and the cotton aphid *Aphis gossypii* [65].

One of the first descriptions of class II HTTs involving a Lepidoptera was reported by Yoshiyama and collaborators in 2001 [66]. Using degenerate primers and PCR, they obtained fragments of a class II *mariner*-like element sharing 98.7% of identity between the parasitoid wasp *Ascogaster reticulatus* and its host, *Adoxophyes honmai* [66]. In another study, the screening of diverse species against a TE database obtained from the sequencing of the twisted-wing parasite *Mengenilla moldrzyki* (Insecta, Strepsitera), revealed that four class II TEs had most likely been horizontally transferred among a huge variety of species: insects, freshwater planarian, hydrozoans, and bats [67], although the ecological network allowing such transfers sometimes seems difficult to trace. Strepsiptera and Lepidoptera diverged over 300 million years ago (MYA), however a member of the *hAT* superfamily (named *Buster1*) found in the Lepidoptera *H. melpomene* and a member of the *CACTA* superfamily (named *spongebob*) in the Lepidoptera *B. mori,* in both cases share more than 87% of nucleotide identity with the elements present in *M. moldrzyski* [67]. In this case, the lepidopteran TEs are defective copies and are no longer autonomous. Another class II TE, the *Helitrons*, is involved in HTTs within Lepidoptera. *Helitrons* were first described in plants and have a particular rolling-circle replication mechanism preserving the original copy in the genome [68]. One *Helitron* appeared to be common in Lepidoptera genomes, since it was detected in more than 30 lepidopteran species, despite the fact that the different copies identified are non-autonomous for transposition [69]. Further analyses have suggested that this widespread distribution could reflect HTTs between lepidopteran species [69]. Moreover, a search for this lepidopteran *Helitrons* sequence in species from other insect orders revealed its presence in aphids, beetles and parasitoid wasps suggesting the occurrence of lepidopteran *Helitrons* HTTs among insects [70]. This last point is consistent with the presence of remnants of lepidopteran *Helitrons* in a bracovirus (Cotesia plutellae bracovirus or CpBV) [71]. In this study, the presence of *Helitrons* in Cotesia sesamiae bracovirus (CsMBV) was also reported, but upon closer examination we found that most of these *Helitrons* remnants are integrated in the vicinity but not within a CsBV proviral segment. Only one *Helitron* (*HeligloriaAii_CpBV1*) is present in a CpBV proviral segment (S22). Zhang and collaborators showed that *Academ*, a recently described class II TE is found in Lepidoptera (*B. mori*, *M. sexta*, *D. plexippus* and *Biston betularia*), and once again in parasitoid wasps (*Cotesia vestalis* and *Microplitis demolitor*), however only one copy of this TE found in *B. mori* seems complete, and is potentially able to transpose [72]. Overall, class II HTTs seem to be frequent among moth and butterflies, since HTTs from the Lepidotera *Spodoptera exigua,* and *Trichoplusia ni* to other lepidopteran species (Bombycidae, Sphingidae, Crambidae, Pyralidae, Papilionidae, Nymphalidae, and Plutellidae) were reported for 19 class II TEs and one class I LTR retroelement [14]. Recently, a global genomic approach, with new TE annotations in available insect genomes, followed by blast search, allowed tracing of 2248 HTTs that have occurred among insects [61]. Concerning the Lepidoptera, HTTs targeted different species: *Amyelois transitella*, *B. mori*, *Calycopis cecrops*, *Chilo suppressalis*, *D. plexippus*, *Heliconius ethilla*, *Heliconius ismenius*, *H. melpomene*, *Heliconius pardalinus*, *Lerema accius*, *Limnephilus lunatus*, *M. sexta*, *M. cinxia*, *Operophtera brumata*, *Papilio machaon, P. glaucus*, *P. polytes*, *P. xuthus*, *Phoebis sennae*, *P. xylostella,* and *Spodoptera frugiperda* [14].

To be effective, HTTs between two species requires a physical shuttle for the transfer of the TE. In order to understand how HTTs could happen, beyond the evidence that they have occurred, Venner and collaborators propose to consider HTTs as a network where the ecological factors take an important place [73]. So far, two main hypotheses are generally proposed. The first posits the use of viruses as TE shuttles. It has recently been shown that baculovirus multiplication in lepidopteran species permits frequent moth TE integration in baculovirus genomes (see bellow) [14]. The second hypothesis relies on host-parasite interactions. In the case of HTTs in Lepidoptera, polydnaviruses that are associated with wasps from the Braconidae family might play an important role, as TEs remnants have been found in their genomes [60,69,71,74,75]. For some of these TEs, HTTs within lepidopteran species are described [66,70,71]. We have proposed several years ago that bracoviruses might act as potential TE shuttles [74]. Indeed, bracovirus proviral segments from which circles are produced have been integrated for millions of years in wasp genomes [17,76], and as such, have been regular targets of TE insertions. Once integrated into a proviral segment, a TE becomes part of the corresponding circle during viral DNA production, and is introduced in the cells of the parasitized host where it could transpose. Provided that it has access to germ cells and that the host survives to parasitism, the TE could be vertically transmitted, continue to transpose and invade the moth genome. Although this is a plausible hypothesis, no convincing evidence has yet been found that would definitively confirm such a role of bracoviruses in HTTs. Finding nearly identical potentially functional TEs shared between a bracovirus and a lepidopteran genome would lift any doubt on this mechanism. Since active transposons are rarely found in genomes, such HTT events might be too rare to be observed in the still fairly limited bracovirus and lepidopteran genome sequence data sets.

## 4. Baculoviruses and Horizontal Transfers in Lepidoptera

Baculoviruses are large circular double-stranded DNA viruses that have been mainly studied in Lepidoptera but are known to infect different insect orders. Two virion morphotypes are involved in the infection process. The occlusion-derived virus (ODV) contains one (granuloviruses) to many (nucleopolyhedroviruses) virions, which are embedded within a protein matrix constituting a polyhedron. ODVs can persist in the environment within polyhedron over several years, and are responsible for the primary infection of lepidopteran larvae within the gut, which occurs while feeding on contaminated leaves. The second form of virions is the budded virus, involved in secondary infections allowing virus spread from cell to cell, which leads ultimately to insect death and tissue liquefaction [77,78]. As baculovirus virions may contain from one to several dozen nucleocapsids, each containing a single genome, baculoviruses should be considered as a population of viral genomes. In this context, genomes with genetic alterations (point mutations, insertions or deletions) can persist within the population and may offer plasticity favoring baculovirus adaptation. Baculoviruses are classified into four genera that differ in their host range, morphological, and genomic features: *Alphabaculovirus* and *Betabaculovirus* that are pathogenic to Lepidoptera, *Gammabaculovirus* to Hymenoptera and *Deltabaculovirus* to Diptera [79]. The most studied baculovirus genus is the *Alphabaculovirus*, for which numerous HTs have been reported over the last four decades, involving in particular, the type species *Autographa californica multiple nucleopolyhedrovirus* (AcMNPV). The HTs that are reported involve mainly transposons, but also genes and their directions are both from insects to baculoviruses and most probably from baculoviruses to insects.

Serial passage of baculovirus in cell culture favors the spontaneous accumulation of genomic alterations among virus genome populations. The presence of TEs, integrated into baculovirus genomes, was first identified based on the study of mutants producing a reduced number of polyhedra (named FP mutants for Few Polyhedra), generated after serial passages (see [80,81] for reviews). FP mutants were shown to produce low occlusion body yields, but achieved high budded virus titers [82,83,84], conferring a selective advantage in cultured cells [85] as there is no need to maintain the ability to infect by feeding using ODVs. The transposable element D (*TED*), which is responsible for such a phenotype, most probably acquired from the *T. ni* host cells, was found to be a class I TE integrated in the AcMNPV *fp* gene [86]. It was more recently classified as an errantivirus (i.e.,: insect retrovirus) having infectious properties [87], and thus having the ability to infect and transpose from one species to another (see [78] for a review).

Recent studies carried out on a larger scale also support the idea that baculoviruses may act as HT vectors from one lepidopteran species to another [14,75]. Indeed, based on ultra-deep DNA sequencing of AcMNPV genome populations, baculoviruses were shown to be exposed *in vivo* to a continuous influx of genetic material from lepidopteran hosts. Sequences transferred between susceptible larvae (*T. ni* or *S. exigua*) and baculovirus genomes comprised a majority (80%) of eukaryotic TEs, the mean insertion frequency ranging from 2.6 to 7.1% [14]. Most of the integrated sequences were shown to occur at particular sites, both at the level of the DNA transposon sequences (at the extremities of the transposon) and at the level of the baculovirus genomes (preferred target sites) (Table 2), as expected in *bona fide* transposition processes [14,75] and as previously observed for FP mutants [88,89,90,91]. In addition to regular transpositions, some HT events might involve other mechanisms, such as homologous recombination, based on micro-homology motifs that were identified between baculovirus and lepidopteran host sequences, or blunt-end ligation [14]. Although integration events seem to be relatively frequent within baculovirus genome populations, and are preferentially found at the level of non-coding regions, they do not appear to be fixed through successive infection cycles, probably due to their deleterious impact on virus fitness. However, they can be maintained when conferring new evolutionary advantages to the virus, such as high budded virus yields in cultured cells in the case of FP mutants.

Once integrated into the genome of a baculovirus, nothing prevents these TEs from being transmitted to a new organism, which can either be another lepidopteran host, or another virus that co-infects the same host, provided that these TEs remain competent to transpose. When this happens during infections allowing the host to survive (for example in a semi- or non-permissive host), an integration that would take place into the host germ line may be vertically transmitted to the next generation. Phylogenetic analyses of *Mariner* and *piggyBack* TE sequences, found in AcMNPV and lepidopteran genomes, revealed lower genetic distance between baculovirus and several lepidopteran sequences than expected given the evolutionary distances of the taxa [14,75]. This showed that *Mariner* and *piggyBack* TEs found in AcMNPV genomes underwent recent HTTs between several species of Lepidoptera [75]. AcMNPV populations, and more generally baculoviruses, were later proposed as potential HTT vectors for a large diversity of TEs and a wide range of lepidopteran families [14].

However, TEs are not the only genetic material that is captured by baculoviruses. Phylogenetic analyses demonstrated several ancient and recent insect cellular gene acquisitions by baculoviruses following HGT [107,108]. Some baculovirus genes, encoding proteins, such as cyclobutane pyrimidine dimer photolyase, protein tyrosine phosphatase, ecdysteroid UDP–glucosyltransferase, acetyltransferase, protein phosphatase 1 regulatory subunit 15A, ribonucleotide reductase small subunit, or transcription terminator factor homologues, are most likely of lepidopteran origin [107,108,109,110,111]. Furthermore, baculoviruses and other large dsDNA viruses, such as entomopoxviruses, were shown to exchange genetic material between themselves [108]. However, despite the growing data available for lepidopteran genomes, to our knowledge, there is no literature demonstrating baculovirus genes might have been captured by Lepidoptera, although such integration events seem quite possible. Traces of viral p74, helicase 2 or helicase homologues can be found in *D. plexippus* or *P. glaucus* genomes using BlastP search against Lepbase (http://blast.lepbase.org/), and the phylogenetic analyses suggest that these genes were possibly acquired from a nudivirus (data not shown). Finally, while it is relatively easy to identify cellular genes acquired by baculoviruses through phylogenetic analyses based on incongruence between gene trees and species trees, it seems much more difficult to identify cellular genes that might be transferred from one species to another, via baculoviruses. This is due to generally low phylogenetic distance between host species, and sometimes is also due to the difficulty of distinguishing putative captured genes from cellular orthologs and paralogs within multigene families.

## 5. Bracoviruses and HGTs in Lepidoptera

Polydnaviruses constitute a unique example in which eukaryotes maintain complex viral machinery in their genome, which provides an essential function for their reproductive success. Polydnaviruses are carried by thousands of species of parasitic wasps that develop within living lepidopteran larvae [112]. The wasp makes use of these viruses to establish successful parasitism in hosts with competent immune defenses [19]. Polydnaviruses, include bracoviruses and ichnoviruses, which are associated with braconid and ichneumonid wasps, respectively. Both groups of viruses mediate the delivery of virulence genes [18] to parasitized hosts, and have evolved by convergence, through at least two genome integration events, involving the insertion of DNA from unrelated viruses into wasp chromosomes [20,21]. Bracoviruses originate from the ancient integration of a nudivirus ∼100 MYA in an ancestor wasp genome [17,76,113,114].

In bracovirus-associated wasps, strikingly, almost all major functions from the ancestor virus (viral transcription, DNA packaging in nucleocapsids, infectivity) have been conserved [17,115,116], except for DNA replication [17,117,118,119] (see [120] for a review). The bracovirus life cycle follows the different steps of a virus cycle, which consists in infecting target cells and producing a viral progeny by making new particles, but these steps are split between the wasp and its parasitized host. Indeed, the particles are produced in the calyx, a specialized region of the wasp ovaries, and the genes involved in this production reside permanently in the wasp genome. Genes involved in viral transcription during an infectious process, such as nudiviral RNA polymerase subunits, are expressed early during wasp pupal development [17,114], and are thought to control the expression of other viral genes, such as those coding for nucleocapsid components, and viral envelope proteins. Endogenous viral sequences are copied and circularized in calyx cells [119,121], the dozens of DNA circles thus produced are packaged into virus particles, themselves secreted in the ovaries, and finally injected into the parasitized host body during wasp oviposition. The particles infect host cells using a conserved set of viral envelope proteins [115] (known in baculoviruses as PIF for *per os* infectivity factors essential for primary infection). The circles present in the particles are released in the nuclei, and the genes they encode are expressed by host cells [120]. These genes produce virulence proteins that are necessary to protect wasp larvae developing within the Lepidoptera from the host immune defenses [122], and that are more broadly involved in altering host physiology and developmental timing. Bracovirus circles were shown to integrate into host cells by using a mechanism involving a specific sequence site termed Host Integration Motif (HIM) [123].

**Figure 2:**
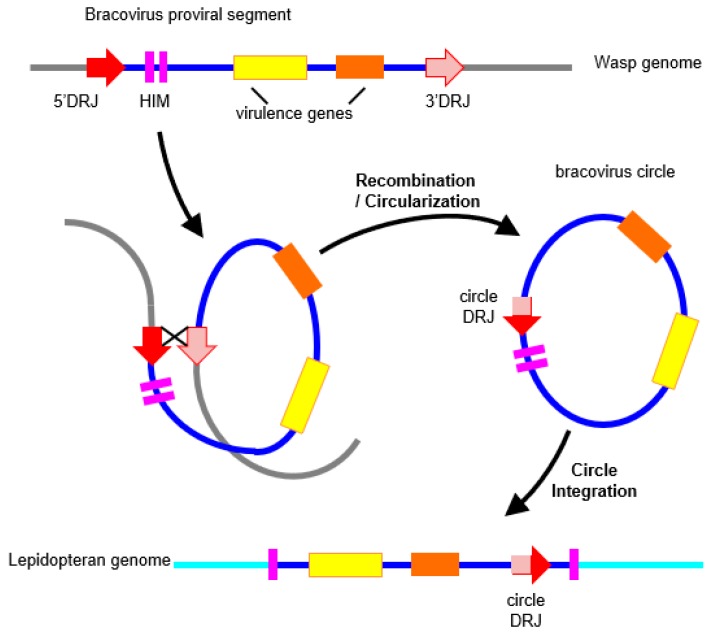
Bracovirus circles are produced from proviral segments present in the wasp genome. Following amplification of proviral segments the produced DNA molecules are circularized by a site-specific recombination mechanism involving slightly different direct repeats corresponding to the extremities of the proviral segments (5’ Direct Repeat Junctions (DRJ) and 3’DRJ: red and pink arrows, respectively). The sequence of the circle contains a circle DRJ (or circle junction motif), which is a recombined form, different from both 5’ and 3’ DRJ (red/pink arrow). Such a recombined DRJ, typical of the bracovirus, has been found in one of the bracovirus insertions in lepidopteran genomes, unambiguously indicating the direction of the DNA transfer was from the bracovirus to the Lepidoptera.

Recently, the search for DNA transfers that are mediated by bracoviruses, revealed that bracoviral sequences were scattered in a variety of lepidopteran genomes, such as those of the monarch (*D. plexippus*), the armyworms (*S. frugiperda and S. exigua*), or the silkmoth (*B. mori*). This was relatively surprising because parasitoids are defined as parasites that kill their host, which are therefore considered as an evolutionary dead-end. These sequences present in lepidopteran genomes can display over 90% similarity at the nucleotide level with bracovirus DNA over several kilobases, which represents an unexpected level of conservation between a lepidoptera and a virus. The largest of such insertions (6.5 kb long), corresponds to more than half of a bracovirus circle sequence, while others are limited to a gene with a few surrounding regulatory sequences [23]. In one case, a regulatory sequence that is involved in bracovirus circle production from amplified viral DNA (Figure 2) is present in the transferred sequence, which unambiguously indicates that the direction of the transfer was from the bracovirus to the Lepidoptera [23]. Integration of bracovirus DNA in the insect genomes was carefully verified, by sequencing junctions between lepidopteran DNA and bracoviral insertions, using either newly collected individuals or individuals obtained from collections. These approaches ensured that chimeric reads did not correspond to assembly errors and were fixed in the species [16]. In addition many short sequences of bracoviral origin (a few hundred base pairs long) were also reported in the monarch and the silkworm genomes based on their detection using bioinformatics screening [124].

**Figure 3:**
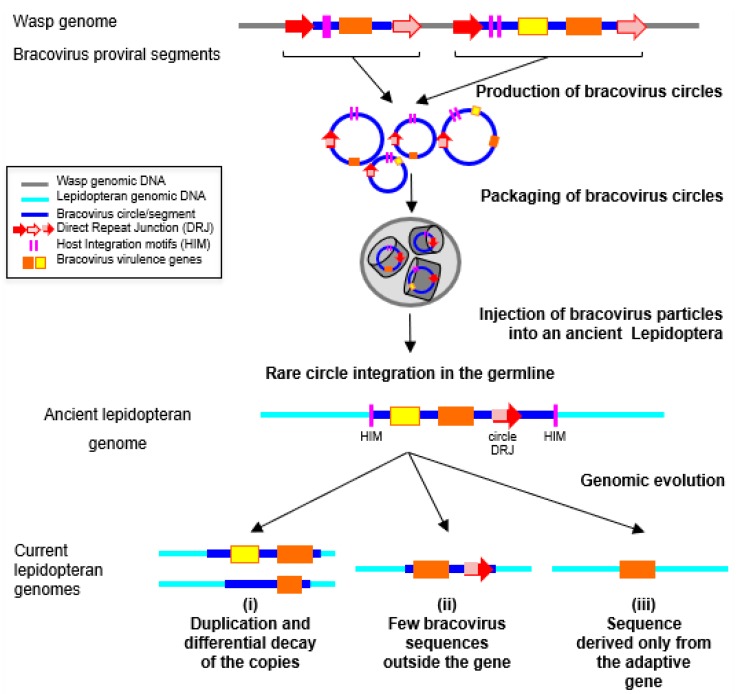
Hypothesis on the process leading to transfer of bracovirus sequences to Lepidoptera genomes, and their evolution once integrated. The bracovirus genome is integrated in the wasp genome (in grey). It is composed of proviral segments (in blue) used to produce dsDNA circles (blue circles), packaged in nucleocapsids (grey cylinders) that encode virulence genes introduced into the host (orange and yellow rectangles), and of bracovirus genes that are involved in particle production (not shown). Direct Repeat Junctions (DRJ, red/pink arrows) are involved in site-specific recombination, allowing circularization of linear molecules amplified from proviral segments (see Figure 2). The circles that are produced are packaged in bracovirus particles that contain several integrase proteins. The particles are injected in the lepidopteran host during wasp oviposition. Once in the host, bracovirus particles infect the cells from virtually all lepidopteran tissues. Moreover, bracovirus circles can integrate into lepidopteran host genomic DNA (in light blue) by a mechanism most likely involving an integrase and is mediated by Host Integration Motifs (HIM), indicated by pink lines. When integration of viral circles occurs in germ cells, they can be transmitted vertically, if the host happens to survive parasitism and reproduce. Since bracovirus genes are adapted for expression in lepidopteran cells, they can be readily domesticated. Once integrated into lepidopteran genomes (“ancient lepidopteran genome”), the bracovirus sequences undergo rearrangements. Different steps in decay of integrated sequences can be observed in the insertions reported. For example, (i) in the monarch genome two copies encoding *ben9* genes are present, likely corresponding to a duplication of an ancestral insertion. The two insertions differ by the presence of a second gene encoding a RnaseT2, and by the length of non-coding sequences of bracovirus origin, the largest copy corresponding to half of the size of the CcBV circle 23. In the case of the BV2-5 encoding insertion in *S. exigua*, (ii) little bracovirus non-coding sequences remain. However, the presence of the DRJ is proof that the sequence originated from a bracovirus (see Figure 2). Ultimately, after original circle integration, only the genes that are domesticated by the Lepidoptera remain, (iii) such as *C-lectin* genes in *Spodoptera*. In this case the bracoviral origin of the gene products was detected by phylogenetic analysis. This figure is mostly based on the life cycle of CcBV associated with *Cotesia congregata* parasitoid wasp of *M. sexta*, except for HIM motifs that have been identified in the bracovirus of *M. demolitor*.

So far, no complete events of circle integration have been detected, although this should be expected in recent integration events, as described in parasitized host cells. Indeed, the integration mechanism involves specific features that correspond to a specific site on the circle, and the loss of around 50 nucleotides [22]. It is noteworthy that these features have allowed detection of several cases of circle re-integration, back in the wasp genome, in different geographic populations of the wasp *Cotesia sesamiae* [125]. The same characteristic features could be used to readily identify recent events of circle integration in lepidopteran genomes, if such integrated circles were available in sequence databases. Thus, although a specific mechanism for circle integration exists, it is not possible to exclude that the bracoviral transferred sequences in lepidopteran genomes might have been integrated through DNA repair mechanisms, based on micro-homologies. This could be the case, in particular, for the short transferred sequences [124]. It is also possible that some insertions could correspond to new types of still uncharacterized transposons, transferring horizontally between Hymenoptera and Lepidoptera. Indeed, before *Polintons*/*Maverick* TEs were characterized, one rearranged copy of these TEs had been detected as a region of Cotesia congregata bracovirus (CcBV) circle 31 sharing an unexpectedly high similarity with nematode and coleopteran sequences [107]. In the case of the monarch, the insertions are ancient since they were found in related species that have diverged over 5 MYA [126]. We supposed that bracovirus insertions correspond to remnants of circles that have been subject to many rearrangements since their integration (Figure 3). Accordingly, one of the insertions is found in two copies, which present different deletions when compared to the related bracovirus sequence.

The presence of bracovirus DNA in Lepidoptera was somewhat surprising because bracovirus injection normally blocks host development at the larval stage, inhibiting metamorphosis [127]. Moreover parasitized hosts most often eventually die, after adult wasp larvae have emerged. In consequence, parasitism should not allow transmission of foreign DNA integrated in the germline. However, it is conceivable that some hosts might successfully defend themselves against the parasite, for example by killing wasps during oviposition, resulting in the reproduction of Lepidoptera that have been infected by a small quantity of bracovirus particles. Moreover, in natural populations, particular biotypes of the parasitoid may not be able to develop within a regular host, as shown in Kenya in the case of *C. sesamiae* parasitizing *Busseola fusca* [128]. On rare occasions, parasitoid wasps could also oviposit in non-host species, and in this case, the virus may not be able to fully interfere with host development [129,130]. In addition, the particles have been shown to enter a large range of insect host cells [123,131], and thus could possibly enter germline cells. We can therefore speculate that bracoviruses may be able to integrate their DNA in germline cells, and in some cases, host development is not blocked, which could allow the stable integration of virus DNA into lepidopteran genomes. These rare events may occur recurrently at evolutionary time scales.

After integration, bracovirus virulence genes [23] may be readily domesticated if their products confer an advantage to the Lepidoptera, since they can be normally transcribed in lepidopteran cells during parasitism. Accordingly, it was shown that bracovirus virulence genes have classical insect gene structures including arthropod transcription start sites and polyadenylation signals [132]. Moreover many gene products display a conserved signal peptide for secretion [132]. More surprisingly, the splicing machinery appears sufficiently conserved between Lepidoptera, to allow correct splicing of bracovirus genes in hosts belonging to different lepidopteran families (such as Noctuidae and Sphingidae) [23]. This capacity may also be important to facilitate the expression of a functional protein, and therefore, favour gene domestication, in the case of bracovirus sequence transfer to a non-host species. Thus, bracovirus genes are already pre-adapted for expression in various Lepidoptera. The genes integrated in the lepidopteran genome can be conserved if they confer a new, advantageous trait for the recipient species.

In the example of bracovirus genes acquired by HGT in the monarch, two copies of *Ben* genes are conserved, expressed, and correctly spliced by the Lepidoptera, but their function is still unknown. *Ben* genes are characterized by the presence of a conserved α-helical module, identified by bioinformatics analyses, which is present in diverse animals and in viruses of two unrelated families (chordopoxviruses and bracoviruses) [133]. In *Drosophila*, a *Ben* gene has been shown to bind to a specific DNA site and to act as a transcriptional repressor of neuronal genes [134]. In CcBV, the *Ben* family comprises thirteen genes, and is the second largest family of virulence factors after the *PTP* (Protein Tyrosine Phosphatase) family [135], suggesting that BEN proteins play an important role during parasitism. Measures of selection pressures operating on these genes in the monarch, and related species, revealed a moderate conservative selection with few sites under positive selection [23], suggesting that these genes may have acquired a function for the monarch. Indeed, useless genomic regions will tend to dissolve in the genome by an accumulation of mutations, recombination, and/or deletion [26,39]. The neighboring regions of the transferred genes will be progressively lost (Figure 2). This process might explain why only partial circles were detected in lepidopteran genomes. But more generally, this is the fate of the majority of HGT events. This could also explain why only a small number of HGTs are eventually detected.

Structural conservation suggests that some transferred genes play a role for the recipient organism. Moreover, functional analyses on transferred genes give insights on their possible role in Lepidoptera. A recombinant baculovirus, producing the transferred bracovirus protein BV2-5, was used to infect lepidopteran cells. Cellular localization by immunofluorescence revealed that BV2-5 had a negative impact on cytoskeleton rearrangement and motility during baculovirus infection, thereby reducing the replication of pathogenic baculovirus in infected lepidopteran cells [23]. However, as found in many cases of resistance, the new properties conferred to cytoskeleton dynamics may also have a detrimental effect in certain circumstances. Indeed, functional analyses of *S. exigua* harbouring *BV2-5* also showed a higher susceptibility to *Bacillus thuringiensis* entomopathogenic bacteria when compared to a European *S. exigua* population that harbours a non-functional truncated version of the gene [23,136]. This suggests that a cost might be associated with the production of BV2-5, resulting in the alteration of cytoskeleton dynamics. Indeed, this might make the larvae more susceptible to other infections, because phagocytosis of bacterial pathogens also involves cytoskeleton dynamics. Altering cytoskeleton dynamics could correspond to the actual BV2-5 function during parasitism. Encapsulation is the main lepidopteran defense mechanism against parasitoid eggs, which involves engulfing a foreign body within a sheath of immune cells. During encapsulation, haemocytes spread and attach on the foreign parasitoid eggs, processes that requires fully functional cystoskeleton. BV2-5 may protect parasitoid eggs by preventing their encapsulation. To note, transcriptomic analysis has shown that *BV2-5* is among the most highly expressed CcBV virulence genes during parasitism of *M. sexta* [132]. This further highlights the potentially important function of the protein in host-parasite interactions. 

The transferred bracovirus *Se-BLL2* gene encoding a C-lectin identified in *Spodoptera* species also had an impact on baculovirus infection. Using purified Se-BLL2 proteins on Sf21 cells infected by baculovirus, and *in vivo* experiments on infected larvae, a dose-dependent negative impact of SE-BLL2 was observed on viral infections [23]. Comparative analysis of three lectins cloned from *S. exigua* showed that SE-BLL2 was efficient to agglutinate cells from a broad range of bacterial species [137]. These results suggest that the transferred bracovirus genes could confer a partial protection to bacterial infections. This could correspond to an immune function of SE-BLL2 during parasitism, as bracovirus associated wasps larvae develop during several days within the parasitized host body. This requires the host quality (regarding pathogen infection) to be maintained, as pathogen infections would result in both host and parasite death.

## 6. Conclusions

The emerging picture is that invertebrate genomes regularly incorporate foreign DNA and that the transferred genes have often been domesticated. Genes acquired by these various HGTs have been maintained because they may represent evolutionary short cuts for the recipient organism to acquire a new function. Ultra-deep sequencing of viral genomes provides new evidence to sustain hypotheses that were formulated years ago that viruses might act as vectors in transfers of TEs or of genes between organisms. 

The genome sequences of many Lepidoptera became available in the past few years, which revealed unexpected findings. The silkmoth, the armyworms, and even the iconic monarch were found to contain highly similar DNA sequences to that of bracoviruses. This DNA likely corresponds to ancient integrations that have undergone rearrangements. These species might thus be considered as naturally produced genetically modified organisms. As bracovirus particles have the ability to integrate into parasitized host DNA, and as the gene structure of the DNA packaged in the particles is already adapted to expression in Lepidoptera, we can suggest that numerous cases of integration and domestication of bracovirus sequences will be identified with the rise of genomic data provided by new generation sequencing. More genes originating from bacteria and fungi will also probably be unveiled in lepidopteran genomes. The challenge is now to determine whether transfers of bracovirus sequences to Lepidoptera occurs frequently or are very rare in natural conditions. Knowing the extent of lateral transfers in ecosystems is important to understand the mechanism and evaluate the risks of diffusion, between different species, of genes that might be introduced in genetically modified organisms.

## Figures and Tables

**Table 1:** Examples of genes from bacteria or fungi acquired by Lepidoptera genomes.

Gene Name	Potential Function/Phenotype	Potential Donor	Lepidopteran Recipient(s)—Genomic or EST Sequences	References
*Glycosyl hydrolase GH31*	Assimilation of plant carbohydrates	Enterococcus bacteria	*B. mori, D. plexippus, P. xylostella, H. melpomene, C. catalpae, A. edwardsi, S. pyri, G. geneura, H. fraternal, L. griseipennis, C. bicoloraria, N. fimetaria, T. erectarius, E. lisa, E. claudia, G. rufobrunnea, P. populi, A. velutinana, A. assama, S. littoralis, M. sexta*	[24,26,27,42,43]
*β-fructofuranosidase GH32*	Assimilation of plant carbohydrates/overcoming plant defense	Bacteria of several genera	*B. mori, D. plexippus*, *H. melpomene, E. postvittana*, *C. fumiferana*, *P. interpunctella*, *O. nubilis*, *B. anynana*, *H. erato*, *T. ni*, *H. virescens*, *S. littoralis, M. sexta*	[24,25,26,42,43,44]
*β-cyanoaline synthase CAS/ cysteine synthase*	Amino acid assimilation/overcoming plant defense	Methylobacterium bacteria	*B. mori, D. plexippus*, *H. melpomene, P. rapae, S. cynthia ricini, M. sexta, S. frugiperda, S. littoralis, T. ni, H. virescens, B. anynana*	[24,26,28,43,47]
*Kynureninase*	Body coloration/amino acid assimilation/overcoming plant defense	*Listeria grayi* bacteria	*B. mori, D. plexippus*, *H. melpomene, C. fumiferana, O. nubilis, B. anynana, H. virescens, M. sexta*	[24,25,26,42,48]
*Glucose-1 phosphatase/inositol phosphatase*	Glycolysis/Resistance to bacterial pathogens	Bacteria of several genera	*B. mori, D. plexippus*, *H. melpomene*, *T. ni*, *H. virescens*, *S. frugiperda, M. sexta*	[24,25,26,42]
*N-methyltryptophane oxidase*	Amino acid degradation/detoxification of malpighian tubules/resistance to bacterial pathogens	Serratia bacteria	*B. mori, M. sexta, A. assama*	[24,25,26,42]
*Glycerophosphoryl diester phosphodiesterase*	Glycerophospholipid metabolism	Pseudomonas bacteria	*B. mori, D. plexippus*, *H. melpomene*, *C. fumiferana, O. nubilis, B. anynana, M. brassicae*, *T. ni, H. virescens, S. littoralis, S. frugiperda, M. sexta, A. assama*	[24,25,26,42]
*Chitinase*	Carbohydrate transport and metabolism	Serratia bacteria	*B. mori, D. plexippus, H. melpomene, C. fumiferana, P. xuthus, B. anynana, H. erato, H. virescens, S. littoralis, S. frugiperda, M. sexta, A. assama*	[24,25,26,42]
*NAD-dependent epimerase/dehydratase*	Nutrient and energy metabolism	Bacteria of several genera	*B. mori, D. plexippus*, *H. melpomene*, *H. virescens*, *S. frugiperda, S. cynthia ricini, A. assama*	[24,25,26]
*Aromatic ring-opening dioxygenase LigB subunit*	unknown	Fungi, or *Naegleria gruberi* amebia	*B. mori, D. plexippus*, *H. melpomene, E. postvittana*, *O. nubilis*, *T. ni*, *H. virescens*, *S. littoralis, S. frugiperda, S. cynthia ricini, A. assama, A. mylitta*	[25,26]
*Alginate lyse/alcohol dehydrogenase*	Energy production and conversion	Bacteria of several genera	*B. mori, D. plexippus*, *H. melpomene*, *E. postvittana*, *C. fumiferana*, *O. nubilis*, *B. anynana, T. ni, H. virescens, S. littoralis, S. frugiperda, S. cynthia ricini, A. assama, A. mylitta, M. sexta*	[25,26,42]
*Gamma-glutamyltranspeptidase*	Amino acid transport and metabolism/resistance to bacterial pathogens	Bacteria of several genera	*B. mori, D. plexippus, H. melpomene, B. anynana, T. ni, H. virescens, S. frugiperda*	[24,25,26]
*Hypothetical protein*	unknown	Lactococcus bacteria	*B. mori, H. melpomene*, *C. fumiferana*, *S. frugiperda, A. mylitta, M. sexta*	[24,25,26,42]
*Methylated DNA-protein-cysteine methyltransferase*	unknown	Bacteria	*D. plexippus*, *H. virescens, S. frugiperda*	[26]

Full name of lepidopteran species and superfamily: *Bombyx mori* (Bombycoidea), *Danaus plexippus* (Papilionoidea), *Plutella xylostella* (Yponomeutoidea), *Heliconius melpomene* (Papilionoidea), *Ceratomia catalpae* (Hesperiidae), *Atrytonopsis edwardsi* (Hesperiidae), *Saturnia pyri* (Saturniidae), *Grammia geneura* (Arctiidae), *Heteranassa fraternal* (Noctuidae), *Lesmone griseipennis* (Noctuidae), *Chloraspilates bicoloraria* (Geometridae), *Narraga fimetaria* (Geometridae), *Tornos erectarius* (Geometridae), *Eurema lisa* (Pieridae), *Euptoieta claudia* (Nymphalidae), *Gonometa rufobrunnea* (Lasiocampidae), *Poecilacampa populi* (Lasiocampidae), *Argyrotaenia velutinana* (Tortricidae), *Spodoptora frugiperda* (Noctuoidea), *Spodoptera littoralis* (Noctuoidea), *Epiphyas postvittana* (Tortricoidea), *Choristoneura fumiferana* (Tortricoidea), *Plodia interpunctella* (Pyraloidea), *Ostrinia nubilis* (Pyraloidea), *Papilio xuthus* (Papilionoidea), *Bicyclus anynana* (Papilionoidea), *Heliconius erato* (Papilionoidea), *Mamestra brassicae* (Noctuoidea), *Trichoplusia ni* (Noctuoidea), *Heliothis virescens* (Noctuoidea), *Manduca sexta* (Bombycoidea), *Samia cynthia ricini* (Bombycoidea), *Antheracea assama* (Bombycoidea), *Antheraea mylitta* (Bombycoidea), *Pieris rapae* (Pieridae).

**Table 2:** Examples of transposable elements from Lepidoptera found in baculoviruses.

TE Type or Name *	Class	Order	Baculovirus	Origin	Preferred Insertion Site	TE Extremities	References
*5S*	I	SINE	AcMNPV	*T. ni*	-	-	[75]
*BEL*	I	LTR	AcMNPV	*T. ni* *S. exigua*	-	-	[14]
*Copia*	I	LTR	AcMNPV	*T. ni* *S. exigua*	-	-	[14]
Gypsy (TED *)	I	LTR	AcMNPV	*T. ni* *S. exigua*	AATG TATAT	-	[14,86,92,93]
*E*	II	TIR	AcMNPV	*S. frugiperda*	TTAA	CCT/AGG	[94]
*Harbinger*	II	TIR	AcMNPV GmMNPV	*T. ni* *S. exigua*	T[A/T]A	GGG/CCC	[14,95]
*hAT*	II	TIR	AcMNPV	*T. ni* *S. exigua*	-	-	[14]
*Helitron*	II	Helitron	AcMNPV	*S. exigua*	-	TC/CTAG	[14,69]
IFP1.6 *	II	TIR	AcMNPV	*S. frugiperda*	TTAA	CCT/AGG	[96,97]
IFP2.2 *	II	-	AcMNPV	*S. frugiperda*	GTTTTTAC	TAC/GTT	[96,97]
*M5*	II	-	AcMNPV	*S. frugiperda*	TTAA	CCG/CGG	[98]
*Tc1-Mariner*	II	TIR	AcMNPV	*T. ni* *S. exigua*	TA	-	[14,75]
			CpGV	*C. leucotreta* *C. pomonella*	TA	CAG/CTG	[99,100,101]
*Mutator*	II	TIR	AcMNPV	*T. ni*	-	-	[14]
*MULE*	II	TIR	AcMNPV	*S. exigua*	-	-	[14]
*P element*	II	TIR	AcMNPV	*T. ni*	-	-	[14]
*PiggyBac (formerly IFP2)*	II	TIR	AcMNPV GmMNPV SeMNPV	*T. ni* *S. exigua*	TTAA	CCC/GGG	[14,75,88,102]
*Sola*	II	TIR	AcMNPV	*T. ni* *S. exigua*	AT	-	[14]
*Tagalong (formerly TFP3)*	II	TIR	AcMNPV GmMNPV	*T. ni*	TTAA	CCC/GGG	[103,104,105,106]
*Transib*	II	TIR	AcMNPV	*T. ni*	CGNCG	-	[14]

Class I: retrotransposon; Class II: DNA transposon; LTR: long terminal repeat; SINE: short interspersed nuclear element, TIR: terminal inverted repeat. Please see [49] for TE classification system. AcMNPV: Autographa californica multiple nucleopolyhedrovirus; CpGV: Cydia pomonella Granulovirus; GmMNPV: Galleria mellonella multiple nucleopolyhedrovirus; SeMNPV: Spodoptera exigua multiple nucleopolyhedrovirus; *T. ni*: *Trichoplusia ni*, *S.exigua*: *Spodoptera exigua*; *S.* *frugiperda*: *Spodoptera frugiperda*; *C.leucotreta*: *Cryptophlebia leucotreta*; *C. pomonella*: *Cydia pomonella*. All baculoviruses listed belong to *Alphabaculovirus* except CpGV (*Betabaculovirus*). *: TE name indicated.
